# The emerging role of cellular senescence in renal diseases

**DOI:** 10.1111/jcmm.14952

**Published:** 2020-01-08

**Authors:** Bingru Zhou, Ying Wan, Rong Chen, Chunmei Zhang, Xuesen Li, Fanyin Meng, Shannon Glaser, Nan Wu, Tianhao Zhou, Siwen Li, Heather Francis, Gianfranco Alpini, Ping Zou

**Affiliations:** ^1^ Department of Pathophysiology Southwest Medical University Luzhou China; ^2^ School of Basic Medical Sciences Institute for Cancer Medicine Southwest Medical University Luzhou China; ^3^ Richard L. Roudebush VA Medical Center Indiana University Indianapolis IN USA; ^4^ Division of Gastroenterology Department of Medicine Indiana University Indianapolis IN USA; ^5^ Division of Gastroenterology and Hepatology Department of Medicine Indiana University School of Medicine Indianapolis IN USA; ^6^ Department of Medical Physiology Texas A&M University College of Medicine Bryan TX USA; ^7^ Department of Physiology Southwest Medical University Luzhou China

**Keywords:** acute renal injury, cellular senescence, diabetic nephropathy, glomerulonephritis, kidney transplanation, renal diseases, renal fibrosis, senescence‐associated secretory phynotype

## Abstract

Cellular senescence represents the state of irreversible cell cycle arrest during cell division. Cellular senescence not only plays a role in diverse biological events such as embryogenesis, tissue regeneration and repair, ageing and tumour occurrence prevention, but it is also involved in many cardiovascular, renal and liver diseases through the senescence‐associated secretory phenotype (SASP). This review summarizes the molecular mechanisms underlying cellular senescence and its possible effects on a variety of renal diseases. We will also discuss the therapeutic approaches based on the regulation of senescent and SASP blockade, which is considered as a promising strategy for the management of renal diseases.

## 1 INTRODUCTION

Cellular senescence refers to an irreversible cell cycle arrest. Senescent cells exhibit a series of changes in cell morphology and epigenetics, including the changes in cell cyclins and increased expression of β‐galactosidase^1^; although there is replicative exhaustion in senescent cells, these cells still have metabolic activity.^1^ The inhibition of DNA replication and rupture of its double‐strand are typical features of cellular senescence.^2^ There are many causes of cellular senescence including genomic damage, activation of oncogenes and inflammation.^3^

Cellular senescence plays a critical role in normal embryonic development in humans and animals and is involved in wound healing.^4^ However, enhanced cellular senescence is also found in multiple organs in the process of ageing or after injury, suggesting that there should be different effects of cellular senescence depending on the pathophysiological context.^5^ More importantly, cellular senescence has been verified as a fundamental cause for the development of many diseases, such as cardiovascular, liver and kidney diseases (Figure 2).^4^ Additional studies confirm that the number of senescent cells increases in multiple anatomical sites in the kidney during ageing and kidney diseases.^5^, ^6^ Therefore, preventing cellular senescence may be a potentially important approach to inhibit the development of chronic kidney disease (CKD).^7^ In this review, we will focus on the mechanisms of cellular senescence and the relationship between senescence and renal diseases.

## 2 GENERAL CONCEPT OF CELLULAR SENESCENCE AND SENESCENCE‐ASSOCIATED SECRETORY PHENOTYPE

In 1961, cellular senescence was first described as a consequence of replicative exhaustion in cultured human fibroblasts.^8^ Senescent cells are alive but display typical features of an enlarged, flattened morphology, senescence‐associated heterochromatin marks, accumulation of lipofuscin granules, expression of β‐galactosidase and lack of mitogenic responses.^1^ Cellular senescence can be classified into two different types according to the presence or the absence of telomere shortening: replicative senescence caused by shortening of telomeres and premature senescence caused by other stress signals such as aberrant oncogene activation and genomic damage.^9^

An important characteristic of senescent cells is their production of a series of proteins named as the senescence‐associated secretory phenotype (SASP).^10^ Through the SASP, senescent cells can affect surrounding cells by secreting various inflammatory cytokines, chemokines, growth factors and extracellular matrix remodelling factors such as interleukin 1α (IL‐1α), interleukin 6 (IL‐6), plasminogen activator inhibitor‐1 (PAI‐1), TGF‐β, connective tissue growth factor (CTGF) and monocyte chemoattractant protein‐1 (MCP‐1).^10^, ^11^ The expression of SASP genes is up‐regulated during senescence, mainly via the actions of NF‐κB and C/EBPβ.^12^ mTOR signalling is also essential for NF‐κB activation and the secretion of pro‐inflammatory SASP genes.^13^ Although the SASP was initially thought to be similar in all senescent cells, the function and specific composition of SASP varies greatly with the different types of stressors, different cell types and environments.

Senescent cells can exert beneficial or harmful effects through SASP and significantly influence their local microenvironment. These SASP factors induce senescence of adjacent cells through a paracrine fashion and contribute to inflammation, which in turn helps to remove senescent cells,^14^ and facilitate tissue repair and remodelling. Some factors promote the tumour‐suppressive function of cellular senescence due to their crucial role for the onset of stable cell cycle arrest.^15^ The benefit of SASP usually exists in a transient situation such as in acute wound‐healing events. However, when senescent cells exist permanently, they may induce serious problems with long‐term harmful consequences due to the chronic secretion of SASP factors such as IL‐1α and MCP‐1. Meanwhile, the increased expression of cytokines and chemokines by senescent cells, such as IL‐6 and MCP‐1, can attract immune cells to improve tissue recovery by secreting more SASP factors and removing harmful factors.^16^ If tissue repair fails, the increased SASP factors may accelerate the process of senescence, ultimately leading to ageing‐associated damage.

## 3 THE MECHANISMS AND RELATED PATHWAYS OF CELLULAR SENESCENCE

### 3.1 Oxidative stress and inflammation

Increasing evidence suggests that persistent DNA damage response triggers cellular senescence.^17^ Oxidative stress can induce DNA damage, which is an important mechanism related to stress‐induced cellular senescence.^17^ Intracellular ROS comes from mitochondria, cell membranes and endoplasmic reticulum. For example, reduction in the nicotinamide‐adenine dinucleotide‐ubiquinone oxidases and dysfunction of the mitochondrial electron transport chain are the main sources of ROS.^18^ Highly reactive oxygen molecules may damage lipids, DNA and proteins. Furthermore, it has been shown that several signalling pathways can be activated by ROS, such as p53/p21 signalling and mitogen‐activated protein kinase pathways, resulting in enhanced apoptosis, inflammation and stress‐induced senescence.^19^ For example, a study has shown that ROS caused telomere‐associated genomic instability and senescence in mesenchymal stem cells.^20^

At the same time, persistent and chronic inflammation may induce senescence, although activation of inflammatory responses is necessary to remove pathogens and mediate tissue repair. For example, several pro‐inflammatory cytokines, especially IL‐6 and IL‐8, are important mediators in the induction of premature senescence. Activation of these two cytokines and their receptors initiates senescence, whereas inhibition of these cytokines blocks senescence.^21^ Also, an intense inflammatory process, including innate and adaptive immune responses, has been shown to regulate kidney senescence.^22^

### 3.2 Increased expression of cyclin‐dependent kinase inhibitors

It is known that cellular senescence is tightly correlated with up‐regulation of cyclin‐dependent kinase (CDKs) inhibitors. The INK4α/ARF locus (the cyclin‐dependent kinase inhibitor 2A (CDKN2A) gene) encodes two different proteins, p16^INK4a^ (hereafter referred as p16) and ARF, through an alternative splicing mechanism. The expression of p16 is primarily regulated by environmental stress‐induced DNA damage,^23^ which is predominant in stress‐induced premature senescence (SIPS). SIPS can be induced by many stress signals, including radiation, oxidative stress, chemical toxicants, DNA damage, oncogenic mutation and nutrient deficiency.^9^ The stimuli‐induced DNA damage response (DDR) is involved in the activation of p16/phosphorylated retinoblastoma (pRb) pathway, especially in epithelial cells.

**Figure 1:**
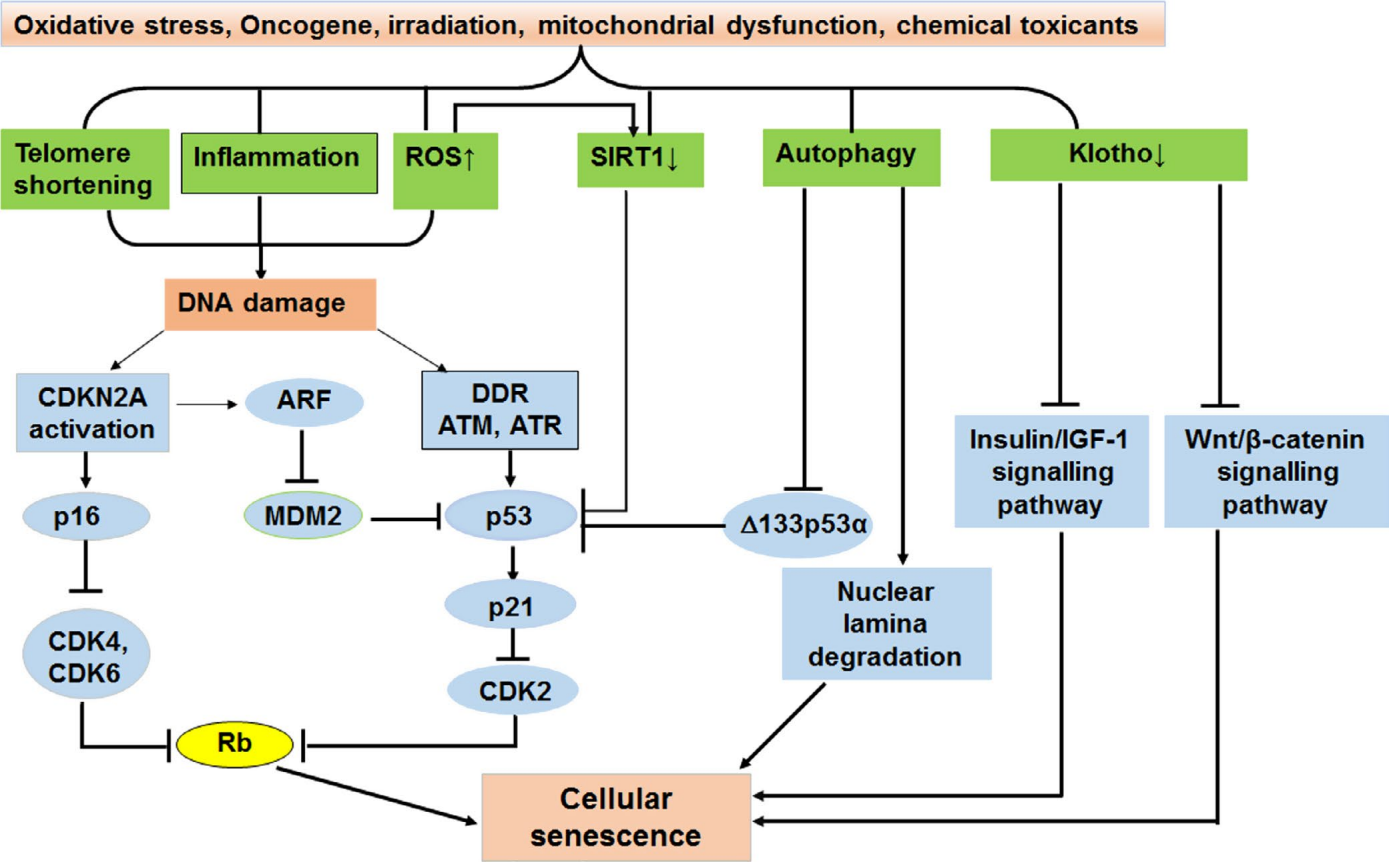
The mechanisms and related pathways of cellular senescence. A variety of stressors induce telomere shortening, increase ROS production, enhance inflammation and autophagy and decrease expression of SIRT1 and Klotho. Telomere shortening, ROS and inflammation usually lead to DNA damage that induces activation of p16/pRb and p53/p21 pathways. Activation of p16 and p21 leads to inhibition of cyclin‐dependent kinase (CDK) complexes and retinoblastoma protein (Rb); thus, senescence is present. Lower expression of SIRT1 causes activation of p53 and induces senescence. Decreased expression of Klotho leads to reduced inhibitory effects on insulin/insulin‐like growth factor‐1(IGF‐1) signalling and Wnt/β‐catenin signalling, resulting in cellular senescence. In addition, autophagy induces cellular senescence through targeting Δ133p53α or lamin B. ROS, reactive oxygen species; DDR, DNA damage response; MDM2, murine double minute 2

The cell cycle inhibitor p16 binds to cyclin‐dependent kinase 4 and 6 (CDK4/6) complex and inhibits its activity, thereby resulting in dephosphorylation of pRb and suppression of G1 phase progression^24^ (Figure 1). The p16/pRb pathway may be essential for the initiation and maintenance of senescence. In addition, the expression of p16 is often used to identify senescent cells due to the expression of p16 in most (though not all) senescent cells.^25^ Therefore, control of p16 expression may be a promising treatment to inhibit cellular senescence.

p53 and p21^Waf1/Cip1^ (hereafter referred as p21) are also CDK inhibitors. Phosphorylation of p53 and enhanced p21 expression are associated with the telomere shortening‐induced senescence and SIPS. p21 can contribute to inhibition of various CDKs, especially CDK2, leading to cell cycle arrest and replicative senescence^26^ (Figure 1). In addition, ARF binds and sequesters the murine double minute 2 oncogene protein into the nucleoli, which leads to inhibition of p53 ubiquitination and degradation, thus resulting in cell cycle arrest through p53/p21 signalling pathway (Figure 1). Evidence strongly suggests that p53/p21 signalling is involved in the initiation of cellular senescence, but p16pRb signalling is necessary to maintain cellular senescence.^27^

### 3.3 Telomere shortening

Telomeres are highly conserved non‐coding repetitive TTAGGG sequences of DNA. These specialized structures protect genetic material from damage by preventing fusion or damage to the chromosome ends. Telomerase is an enzyme that maintains telomeres and sustains chromosomal stability. As there is no telomerase expression in most human cells, except in the cells with highly proliferative capacity (bone marrow, skin and germ cells),^28^ telomeres shorten progressively as cells divide. Telomere shortening causes genetic instability and cell apoptosis, and is the main trigger of replicative senescence.^29^ Replicative senescence is found in many kinds of cells such as fibroblast, keratinocytes, endothelial cells, lymphocytes, adrenocortical cells and chondrocytes.^30^ In multiple cells, telomeres shorten progressively with successive cell division. In addition to replicative senescence, cellular senescence induced by various harmful stimuli can also result in shortening of the telomeres.^29^ A persistent DDR is activated when telomeres shorten to a critical length, which leads to the down‐regulation of genes involved in cell cycle progression and the up‐regulation of growth suppressors such as p53 and p16 (Figure 1).

### 3.4 Down‐regulation of Klotho or Sirtuin 1 expression

In 1997, Klotho was discovered as a transmembrane protein usually expressed in proximal and distal renal tubules, which was subsequently identified as an anti‐ageing factor and shown to exert inhibitory effects on insulin/insulin‐like growth factor‐1 signalling (Figure 1), oxygen free radicals and phosphate/calcium homoeostasis.^31^ Multiple ageing‐associated diseases develop in mice with genetic deficiency of Klotho, including osteoporosis, stroke and arteriosclerosis. Conversely, mice with overexpression of Klotho gene exhibit longer lifespan.^32^ If Klotho is exogenously administered, the senescence of endothelial cell can be significantly reduced.^33^ Interestingly, the levels of Klotho were significantly decreased in old patients (80‐89 years) with CKD compared to patients with CKD at the age of 60‐69; serum Klotho levels were inversely correlated with age, suggesting that Klotho may serve a biomarker of ageing progression.^34^

Sirtuin 1(SIRT1) is also recognized as a key modulator of ageing. SIRT1 can deacetylate histone as well as non‐histone proteins such as FOXOs, p53 and NF‐κB,^35^, ^36^ thereby influencing several important signalling pathways associated with cellular stress, metabolism and longevity. It has been shown that SIRT1 can inhibit cellular senescence of endothelial cells.^37^ Down‐regulation of SIRT1 in endothelial cells leads to premature senescence‐like phenotype by enhancing acetylation of p53. On the contrary, increased expression and activity of SIRT1 prevent cells from entering into the state of senescence by inhibiting p53 activity.^37^ Furthermore, it has been shown that inhibition of miR‐570‐3p in small airway epithelial cells from COPD patients restores SIRT1 expression, leading to SIRT1‐dependent inhibition of senescence markers and cellular rejuvenation.^38^

### 3.5 Other mechanisms

Another possible mechanism involved in cellular senescence is autophagy. Autophagy is a key catabolic cellular process, which degrades damaged proteins as well as organelles. Autophagy often exerts protective effects under various stress conditions.^39^ Recently, autophagy was considered as an effective mechanism involved in the induction of cellular senescence.^40^ During senescence, autophagy is induced and activated, consequently facilitating the process of senescence. In addition, the senescence phenotype could be delayed if autophagy is inhibited.^41^ Furthermore, autophagy can cause cellular senescence by targeting Δ133p53α, a p53 isoform that inhibit full‐length p53, or contributing to nuclear lamina degradation under various circumstances^42^, ^43^(Figure 1).

Senescent cells have been shown to be resistant to apoptosis.^44^ The BCL‐2 protein family, including the anti‐apoptotic proteins BCL‐2, BCL‐W and BCL‐XL, is essential for senescent cell resistance to apoptosis.^45^ Increased expression of BCL‐W and BCL‐XL is involved in senescent cell resistance to apoptosis, and inhibition of BCL‐W and BCL‐XL causes death of senescent cells. Furthermore, a small‐molecule inhibitor targeting the BCL‐2, BCL‐W and BCL‐XL proteins (ABT‐737) contributed to apoptosis of senescent cells both in vivo and in vitro.^45^

In addition, Wnt/β‐catenin signalling is involved in the pathogenesis of cellular senescence (Figure 1). Wnt/β‐catenin signalling is a conserved signalling pathway in organ development that remains silent in normal adult kidneys.^46^ According to a recent study,^47^ Wnt/β‐catenin signalling and renin‐angiotensin system (RAS) activity were up‐regulated in ageing kidneys. Moreover, inhibition of Wnt/β‐catenin signalling significantly protected the normal structure and function of mitochondria, which reduced age‐related renal fibrosis. At the same time, ectopic expression of Klotho, an antagonist of endogenous Wnt/β‐catenin activity, eliminated renal fibrosis in d‐lactose‐induced accelerated ageing mouse model by alleviating cellular senescence and mitochondrial dysfunction.^47^

## 4 MARKERS FOR DETECTION OF SENESCENCE

Currently, it is difficult to find unique markers to identify and quantify senescent cells, especially under in vivo conditions. Senescent cells are classically arrested at G1/S transition of the cell cycle triggered by eroded telomeres in ageing cells, thus typically display a DNA content characteristic of the G1 phase and express distinct cell cycle inhibitors such as p16, p21 and p53.^48^ Subsequently, p21 has been shown to mediate permanent cell arrest in G2/M transition induced by DNA damage through inhibiting mitotic CDK complexes and pRb phosphorylation.^49^ Assessment of these cell cycle inhibitor expression is very helpful for senescence evaluation.

In addition to aforementioned CDK inhibitors, other well‐known phenotypic markers are also considered to be indicators of cellular senescence. The secretion of SASP factors and the mitochondrial dysfunction^50^ are consequences of senescence**,** which stabilize the senescent state and promote paracrine senescence of neighbouring cells. Therefore, monitoring the alterations of mitochondrial dynamics and the secretome may be used to estimate senescent cell burden. Increased expression and activity of senescence‐associated β‐galactosidase (SA‐β‐gal, a lysosomal hydrolase) are commonly applied to identify senescent cells.^51^ Also, telomere shortening inducing replicative limits can also indirectly, but effectively, identify cellular senescence. Increased production of senescence‐associated DNA damage foci (SADF) and senescence‐associated heterochromatic foci (SAHF) in the nucleus are also regarded as senescence markers. SADFs contain important proteins for induction of senescence.^52^ Overexpression of proteins related to SAHF formation can also induce senescence and inhibit the expression of genes associated with proliferation.^53^ Another common senescence marker is γ‐H2AX, which is produced by the phosphorylation of the histone H2AX. Additionally, senescent cells often demonstrate distinctive changes in nuclear morphology associated with loss of lamin B1 (Figure 1), which is also a senescence‐associated biomarker.^54^ In fact, until now, there is no single marker can be used to entirely define cellular senescence. Therefore, aforementioned markers have been used together to determine senescent cells in different disease and ageing contexts.

In addition, DcR2, a decoy receptor for tumour necrosis factor‐related apoptosis‐inducing ligand, is a marker of senescence.^55^ Previous studies found that DcR2 is highly expressed in senescent tumour cells and senescent hepatic stellate cell.^55^ Recently, it has been shown that DcR2 is exclusively expressed in renal tubular epithelia and co‐expressed with senescent markers in patients with diabetic nephropathy, and the extracellular portion of DcR2 is detectable in urine.^56^

## 5 SENESCENCE IN RENAL DISEASES

### 5.1 Type of renal senescent cells

During ageing and renal diseases, senescent cells can be observed in the cortex and medulla. Generally, proximal tubular cells in the cortex are the majority of senescent cells. They are predominant G2‐arrested senescent cells.^57^ Although senescent cells are mainly present in tubular epithelial cells, other kinds of cells may become senescent. For example, increased p16 expression was described in the glomerular cells during glomerular diseases, such as glomerulonephritis, membranous nephropathy, diabetic nephropathy and focal segment glomerular sclerosis.^58^, ^59^ In addition, vascular and interstitial cells may undergo senescence during glomerular diseases and hypertension.^58^ Therefore, different stressors that occur on different site may decide the localization and type of senescent cells.

**Figure 2:**
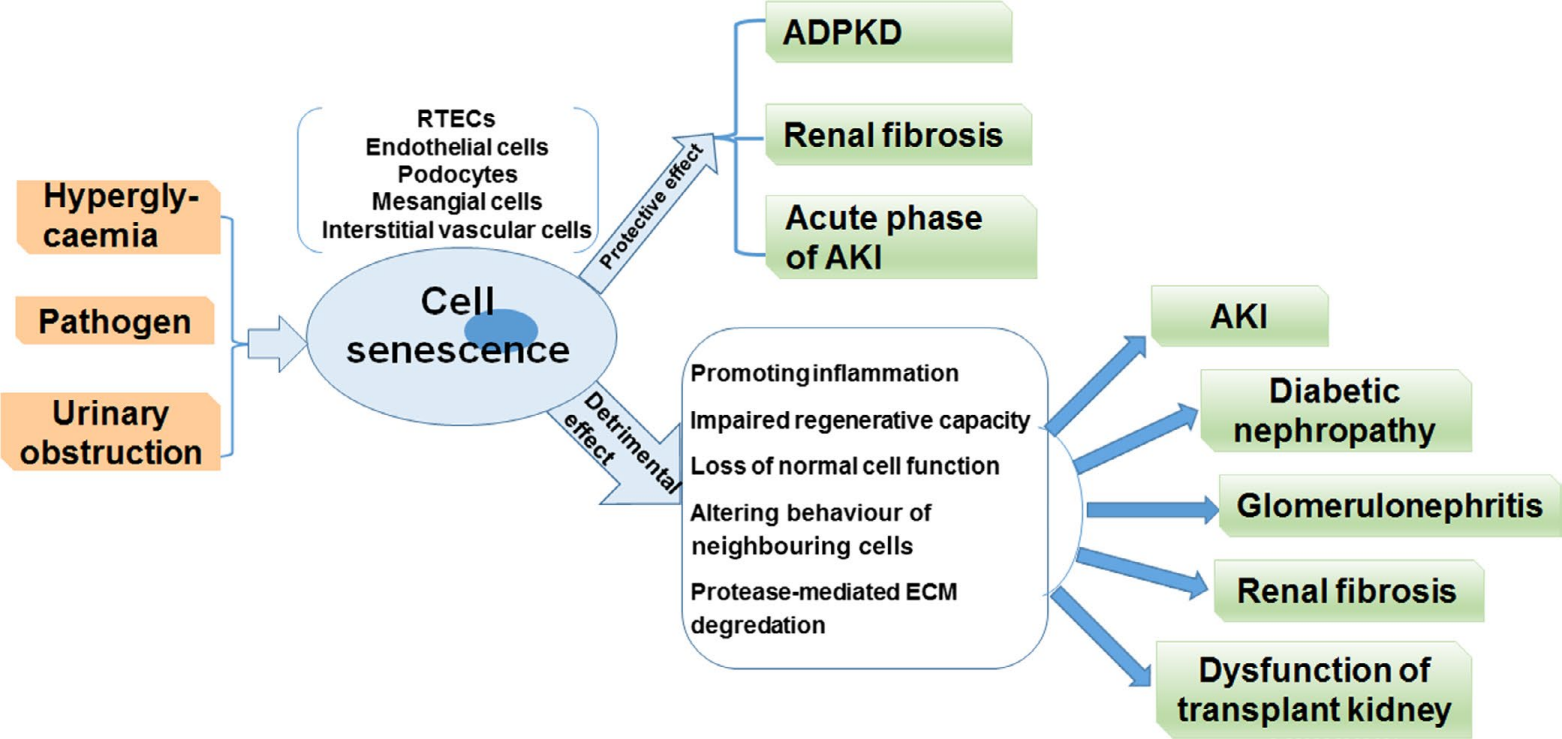
A schematic view of the effects of cellular senescence in renal diseases. In renal diseases, various types of cells may experience senescence such as endothelial cells and renal tubular epithelial cells (RTECs). Although senescence may have protective effects in the development of autosomal dominant polycystic kidney disease (ADPKD), acute phase of acute kidney disease (AKI) and renal fibrosis, senescence can promote the progression of several renal diseases, including AKI, diabetic nephropathy, glomerulonephritis, renal fibrosis and dysfunction of transplant kidney

In the progression of kidney diseases, various types of cells, including renal tubular epithelial cells, podocytes, endothelial cells, interstitial cells, immune cells and mesangial cells (Figure 2), may become senescent and secrete a lot of factors, which are collectively named the CASP (CKD‐associated secretory phenotype).^57^ SASP and CASP share many similarities, which might be the mediator of the crosstalk between cellular senescence and CKD.^57^

### 5.2 Acute renal injury

Acute renal injury (AKI) is the primary driver of renal injury and CKD. AKI is characterized by a sudden deterioration of kidney function and high morbidity and mortality. During AKI, the kidneys fail to eliminate metabolic wastes, concentrate the urine and maintain water and electrolyte balance.^60^ Due to a rise in the elderly population, the incidence of AKI has significantly increased in recent years because age‐related renal functional and morphological changes as well as accompanying comorbidities may promote the vulnerability of the elderly population to AKI.^61^ Increased susceptibility to injury and lack of sufficient repair in ageing and ischaemic kidneys may be associated with telomere shortening and other senescence‐related mechanisms.^62^

Cell cycle arrest may serve as a protective mechanism following AKI by allowing the cell to avoid the replication of damaged DNA. A study described that p21 knockout mice were more susceptible to ischaemia‐induced acute renal failure.^63^ On the other hand, it has been shown that mitotic arrest at the G2/M phase plays a crucial role in response to AKI, where it drives maladaptive repair and progressive fibrosis; this suggests that renal recovery after injury would be hampered due to increased numbers of senescent cells and reduced regenerative capacity of aged kidneys. Senescent tubular cells will reduce cell proliferation for repairing injury and contribute to the progression of renal fibrosis.^64^ A study demonstrated that sustained epithelial Notch activation may trigger a pro‐senescent state and maladaptive repair after ischaemia/reperfusion damage, leading to more inferior outcome of old kidneys after injury.^65^ Therefore, the role of cell senescence in AKI is complex which needs to be elucidated in different time points of AKI or different models of AKI.

### 5.3 Diabetic nephropathy

Diabetic nephropathy is a major cause of end‐stage nephropathy. During the development of diabetic nephropathy, proteinuria is positively correlated with high‐glucose‐induced glomerular cell senescence, which is related to the increased production of ROS.^66^ A study demonstrated that proximal tubule epithelial cells (PTECs) are the major target for impaired glucose‐induced metabolic disorders.^67^ Also, Tsai et al confirmed that high glucose can induce the senescence of PTEC by increasing the expression of microRNA‐378i, a potential biomarker of kidney damage in diabetic nephropathy.^68^ Meanwhile, kidney interstitial injury is also critical to the development of diabetic nephropathy.^66^

Increased p16 expression and SA‑β‑gal activity were observed in tubule cells, mesangial cells, podocytes and endothelial cells from patients with type 2 diabetic nephropathy^59^ and mice with streptozotocin‐induced type 1 diabetes mellitus.^69^ Furthermore, several studies have demonstrated a direct link between hyperglycaemia and the induction of senescence in vitro in cultured proximal tubule cells^69^ and mesangial cells^70^ as well as in vivo in a mouse model of type 1 diabetes mellitus.^69^ Overall, these results indicate that hyperglycaemia is a crucial driver of cellular senescence, which may contribute to the progression of diabetic nephropathy.

### 5.4 Glomerulonephritis

Recent studies show that cellular senescence is closely related to glomerular lesions and the degree of glomerular ageing is also related to the progression of nephropathy.^58^ For example, during IgA nephropathy (IgAN), disease progression is related to telomere shortening^71^ as well as several other senescence‐related alterations such as elevated SA‑β‑gal activity and increased expression of p16 and p21.^72^ These findings suggest that cellular senescence may play a crucial role in the development of IgAN. In this case, however, it remains to be clarified whether senescence is only associated with tissue damage or whether it promotes disease progression. Through a series of experiments and tests, Chen et al found that mice with lupus nephropathy (LN) and severe proteinuria showed enhanced SA‐β‐gal expression and administration of dexamethasone (DEX) reduced SA‐β‐gal expression and renal dysfunction, which suggested that accelerated senescence of the glomerulus was related to the development of LN.^73^ Another study showed that bone marrow (BM)‐derived mesenchymal stem cells (BM‐MSCs) exhibited signs of senescence in systemic lupus erythematosus (SLE) patients and MRL/lpr mice. The senescent phenotype of BM‐MSCs was involved in the pathogenesis of LN in MRL/lpr mice, which can be reversed by rapamycin.^74^ In addition, Chuang et al found that the reduction in podocyte SIRT1 led to senescence of podocytes along with reduced activation of peroxisome proliferator‐activated receptor(PPAR)‐a coactivator‐1(PGC1a)/PPAR‐γ, forkhead box O (FOXO)3, FOXO4 and p65 NF‐κB, resulting in aggravated ageing‐induced glomerulosclerosis.^75^

### 5.5 Renal fibrosis

Renal fibrosis is common in end‐stage renal failure and may develop from a variety of kidney diseases. Eventually, renal fibrosis leads to loss of kidney function. Recent studies indicate that the kidney with renal fibrosis is more prone to develop CKD due to incomplete recovery after AKI.^76^ Health regeneration or recovery after AKI should result in recovery of normal structure with differentiated tubule epithelium. However, due to severe damage, recovery of normal structure is often incomplete which contributes to the development of focal tubulointerstitial fibrosis.^76^ Renal fibrosis may be caused by the accelerated senescence of tubular cells; for example, Wnt9a induced senescent tubular cells to produce TGF‐β1, driving proliferation and activation of rat kidney fibroblasts.^77^ In addition, TGF‐β1 may induce premature senescence of mesangial cells and myofibroblast‐like phenotype transformation, which may contribute to development of glomerulosclerosis.^78^ At the same time, it was reported that G2/M‐arrested proximal tubular cells activated c‐jun NH(2)‐terminal kinase (JNK) signalling, promoting pro‐fibrotic cytokine production.^79^ The inactivation of p16 alleviated nephron atrophy and interstitial fibrosis in kidney transplant experiments.^2^ Therefore, senescent cells play a crucial part in the development of renal fibrosis.

The role of cellular senescence in renal interstitial fibrosis appears to be complex. Senescent cells may contribute to pro‐fibrotic circumstances through their SASP in ageing and kidney diseases.^16^ But the specific role of cellular senescence in renal fibrosis has not yet been elucidated. Contrary to the general opinion about the detrimental effect of cellular senescence, there is evidence regarding a beneficial role of senescence in renal fibrosis. It has been reported that p16 inactivation promoted renal interstitial fibrosis in normal mice and a mouse model of unilateral ureteral occlusion.^80^ These seemingly contradictory findings demonstrated dual role of cellular senescence in different kidney injury processes. Further studies are needed to elucidate whether different effector molecules are involved in the action of senescent renal tubular cells.

### 5.6 Kidney transplantation

CKD as a trend in a variety of kidney diseases can lead to end‐stage renal disease (ESRD) if the renal function gradually deteriorates. In this case, kidney function deteriorates and alternative therapies such as a kidney transplant are necessary for patients with ESRD.^81^ Elderly patients are more susceptible to the complications of kidney transplantation and the side effects of the immunosuppressive regimen, showing a lower survival rate after transplant.^82^ Cells from various tissue of the human body become senescent with age; therefore, ageing may also be one of the factors to affect the therapeutic effect of kidney transplantation. Other studies have demonstrated that ischaemia‐reperfusion injury during kidney transplantation causes oxidative stress, which in turn induces cellular senescence and accelerates dysfunction of the donor organ.^83^, ^84^ In experimental rat models, kidney transplant resulted in transient elevation of p21, sustained increase in p16, enhanced expression of SA‐β‐gal expression and accelerated telomere shortening.^85^ Furthermore, other studies showed that mice undergoing kidney transplant coupled with a loss of INK4a had a significantly better survival rate, less interstitial fibrosis and improved tubular cell proliferation, compared with mice that received a wild‐type transplant.^2^ Although kidney transplantation is the preferred treatment for ESRD,^86^ long‐term failure of kidney transplants remains an important clinical problem. Long‐term graft survival is affected by donor age. Ageing increases AKI and reduces renal regeneration capacity. Further, interstitial fibrosis and tubular atrophy were observed in ageing kidney graft. Therefore, kidney transplant senescence could promote graft loss. In addition, in human kidney transplant, CDKN2A expression in implantation biopsies was related to donor age and graft function.^87^ Telomere length assessed in biopsies collected in the peri‐transplant period can predict the long‐term kidney allograft function, and a significant shortening of telomere was observed in patients with delayed graft function, acute rejection and chronic allograft dysfunction.^88^ Therefore, all these findings demonstrated that senescence plays a crucial role in kidney transplantation, which may affect the outcome and prognosis of kidney transplant.

### 5.7 Polycystic kidney disease

The most common kind of polycystic kidney disease (PKD) is autosomal dominant polycystic kidney disease (ADPKD), which leads to ESRD in patients between 50 and 70 years.^89^ PKD belongs to the ciliopathies family and mainly influence the ciliated epithelial cells that line the renal tubules. PKD can also lead to tubule dilation by increasing cell proliferation.^90^ Reduced p21 levels in the kidney are found in a rat model of PKD and patients with ADPKD.^91^ Additionally, application of a CDK inhibitor, roscovitine, increases SA‑β‑gal activity, restores p21 expression, reduces proliferation of renal tubular cells and slows disease progression in an ADPKD mouse model.^92^ In contrast to aforementioned renal injury, these findings demonstrate that cellular senescence attenuates ADPKD progression.

## 6 THERAPEUTIC INTERVENTIONS FOR RENAL DISEASES BY REGULATING SENESCENCE

Due to the important role of senescence and SASP in kidney disease, they have been proposed to be therapeutic targets for the treatment of these diseases. It has been verified that some drugs (including glucocorticoids, rapamycin and resveratrol) have extensive pharmacological effects in the management of renal diseases. Notably, these drugs have been shown to reduce ROS levels and transcriptional activity of NF‐κB, thereby decreasing cellular senescence.

For instance, rapamycin reduces transcription and translation of SASP factors, eventually resulting in secretome depletion through inhibition of mTOR and NF‐κB transcriptional activity mediated by suppression of IL1A translation.^13^

Similar to the effects of rapamycin, pre‐treatment with dexmedetomidine also decreased the number of senescent tubular cells and weakened the protein expression of p53, p21 and p16,^7^ thus alleviating renal ischaemia/reperfusion‐induced AKI and chronic renal fibrosis in later stages.

The important role of senescence in multiple diseases contributes to the exploration of novel treatments that can regulate senescence. To overcome the detrimental effects of senescence on renal injury and ageing, the pharmacological effect of these novel treatments should focus on reducing the accumulation of senescent cells or increasing the clearance of those cells. Anti‐senescence compounds mainly include senolytics or senotherapeutics. Studies from animal experiments demonstrated that these compounds reversed ageing phenotypes and improved kidney function.^93^ ABT‐263(navitoclax), an inhibitor of Bcl‐2 family, has been confirmed as a senolytic, showing to remove senescent cells in aged mice and allow for tissue regeneration.^94^ However, the side effect of navitoclax lies in apoptosis and is not restricted to cellular senescence. Other BCL‐2 family inhibitors such as A1331852 and A1155463 were manifested to be toxic and did not target all senescent cells.^95^ Therefore, the benefit of removing senescent cells has not been completely clarified by these experiments. Feasibility of this treatment for each kidney disease should also be evaluated**.**

Kidney transplantation may be a suitable scenario for the application of senolysis. Anti‐senescent treatments should focus on the removal of sustained senescent cells but not on preventing the induction of a temporary cell cycle arrest at the early stage of kidney transplantation. The anti‐senescence compound FOXO4‐DRI has proven to be potent for therapy of the ageing kidney. In senescent cells, FOXO4 was observed to serve as a binding partner of p53, preventing these cells from p53‐induced pro‐apoptotic response. Therefore, FOXO4‐DRI can interrupt FOXO4‐p53 interaction and contribute to p53‐induced apoptosis of senescent cells.^96^

In addition, chronic treatment of the senolytic drug quercetin alleviated the expression of p16, p19 and p53, reducing renal fibrosis induced by obesity and dyslipidaemia.^97^ Interestingly, Leung et al found that the treatment of YAP inhibitor verteporfin inhibited activation of senescence‐associated genes, SASPs and activation of Stat3 as well as impeded the development of tubulointerstitial fibrosis.^98^ Furthermore, erythropoietin can preserve tubular epithelial cell regeneration and reduce renal fibrosis in mice with unilateral ureteral obstruction by inhibiting SIPS and epithelial‐to‐mesenchymal transition of renal epithelial cells.^99^

For diabetic nephropathy, it has been shown that rapamycin or resveratrol inhibits mesangial cells (MCs) senescence induced by high glucose through increasing the SIRT1 expression and activity, which was completely blocked by treatment with siRNA‐SIRT1.^100^

In addition, as mentioned, in an experimental LN study, intragastric administration of rapamycin alleviated symptoms of LN by reversing the senescence of BM‐MSCs in MRL/lpr mice through inhibition of the mTOR signalling pathway.^74^ Also, CDK inhibitors such as roscovitine may be a therapeutic approach for the treatment of PKD.^92^

## 7 SUMMARY/FUTURE PERSPECTIVES

Overall, cellular senescence and SASP are involved in kidney injury during both degenerative and hyperplastic diseases. In many renal diseases and in the ageing kidney, there is an accumulation of G1‐ and G2‐arrested senescent cells in multiple regions within the kidney, especially in the renal tubular cells of the cortex. Thus, renal function in failing or ageing kidneys may be improved by targeting senescent cells or SASP.

During the development of renal disease, senescence plays a time‐dependent role in the progression of renal fibrosis, which is beneficial in the early stage but detrimental in the late stage with long‐term adverse consequences. Therefore, it is important to understand how cellular senescence plays a protective or a detrimental effect during different periods of renal injury and the mechanisms of senescence underlying the progression of CKD such as renal fibrosis. Further research is needed to clarify the characteristics of all senescent cells and the specific targets of senescent cells in renal diseases. Until now**,** most findings were acquired through studying animal models; therefore, the role of senescence in human renal diseases should be validated in future studies. Additionally, novel strategies to inhibit the progression of CKD by removing senescent cells in vivo, including targeted gene therapy to avoid side effects in other organs, need further exploration.

## CONFLICT OF INTEREST

All authors declare that they have no conflicts of interests.

## AUTHORS’ CONTRIBUTIONS

GA and PZ developed and organized this paper; YW and BZ mainly drafted the paper and created the figures. All other authors participated in revising the paper and finalizing the paper. All authors gave approval for publication.
